# The relationship between physical activity and trait anxiety in college students: The mediating role of executive function

**DOI:** 10.3389/fnhum.2022.1009540

**Published:** 2022-09-23

**Authors:** Zhiwei Dong, Peng Wang, Xin Xin, Shufan Li, Jing Wang, Jinlei Zhao, Xing Wang

**Affiliations:** ^1^School of Physical Education and Training, Shanghai University of Sport, Shanghai, China; ^2^School of Physical Education, Lixin University of Accounting and Finance, Shanghai, China

**Keywords:** physical activity, college students, trait anxiety, executive function, mediating effects

## Abstract

**Objectives:**

Aimed to analyze the mediating effect of executive function between physical activity level and trait anxiety in college students.

**Methods:**

The International Physical Activity Questionnaire, State-Trait Anxiety Inventory, Stroop task, 1-back task, and More-odd shifting task were used to analyze 248 college students.

**Results:**

Trait anxiety were significantly correlated with shifting function (*r* = 0.182, *P* = 0.004) and inhibition function (*r* = 0.163, *P* = 0.010) and not with working memory (*r* = 0.056, *P* = 0.385). Vigorous physical activity (VPA) was most highly correlated with inhibition function (Beta = −0.144, *P* = 0.024) and working memory (Beta = −0.208, *P* = 0.001), and light physical activity (LPA) was most highly correlated with shifting function (Beta = −0.211, *P* = 0.001). Physical activity had a 72.31% association with trait anxiety (B = −0.195), with 11.79% mediated by inhibition function (B = −0.023) and 15.90% by shifting function (B = −0.031).

**Conclusion:**

College students' physical activity promotes both inhibition and shifting functions, which in turn affect trait anxiety. VPA had a direct effect, while the effect of moderate physical activity (MPA) and LPA was completely mediated exclusively through executive functions, and the mediating effect of shifting function was the highest. It is recommended that college workers should motivate students with high trait anxiety to engage in more VPA and pay attention to changes in their inhibition and shifting functions.

## Introduction

Anxiety is an individual's emotional experience of worry, annoyance, and uneasiness about existing or potentially threatening situations, often accompanied by irritability, somatic tension, and sleep disturbances (Norbury and Evans, 2019; Qi et al., 2019). It is reported that 11.11~39.88% of college students suffer from the invasion of anxiety (Mu et al., 2019; Ramón-Arbués et al., 2020; Zhao et al., 2021), 48.74%~49.04% of college students with anxiety symptoms have self-injurious behaviors (Zhao et al., 2021; Li, 2022), and the high correlation between anxiety and suicidal ideation has become another high-risk factor for suicide in addition to depression (Luo et al., 2021). Anxiety is divided into state anxiety and trait anxiety. Trait anxiety is a relatively stable and individualized anxiety tendency over a period of time (Marteau and Bekker, 1992). Compared with state anxiety, trait anxiety is a more dangerous condition, especially for individuals with high trait anxiety, who are more vulnerable to anxiety disorders, depression, and Alzheimer's disease under stressful situations (de Bles et al., 2019; Li et al., 2021), and are of great concern to researchers and college administrators.

Available research suggests that anxiety is associated with poorer executive function. Executive function is a high-level cognitive process that controls and regulates other cognitive processes during complex cognitive tasks (Funahashi, 2001), and contains three core subcomponents: inhibition, shifting and working memory (Miyake et al., 2000). Executive function and emotional state mutually influence each other, with positive emotional state promoting high levels of cognitive regulation and, in turn, higher executive function helping individuals to cope with reality dilemmas reasonably and efficiently and reducing negative emotions (Wang and Wang, 2007). Thus, higher executive function is often associated with positive emotions. Individuals with high trait anxiety have longer reaction times and higher error rates on inhibition tasks (Yu et al., 2018), struggle more to inhibit distracting information (Bai et al., 2016) and have longer N2 latencies and higher wave amplitudes (Shields et al., 2016). They also react longer and have higher error rates on shifting tasks (Ansari et al., 2008), and as task complexity increases, the disadvantage of shifting function becomes more pronounced (Moran, 2016). Anxiety levels also correlate negatively with performance on tasks such as n-back and verbal-visual-spatial memory (Chen et al., 2013).

Physical activity is an effective means of relieving negative emotions such as anxiety, with the advantages of high compliance, low side effects, and stable outcome (Hallgren et al., 2017). Several Meta-analyses have shown that regular exercise for more than 2 weeks can significantly reduce anxiety symptoms in people of different ages (Aylett et al., 2018; Kazeminia et al., 2020; Ramos-Sanchez et al., 2021). In addition, physical activity may also indirectly improve mood by enhancing executive function in two main biological hypotheses. The first, physical activity increases individual arousal levels, enhances cerebral blood perfusion (Klein et al., 2019), and promotes brain structure, especially gray matter integrity in the prefrontal and medial temporal regions (Hou et al., 2020), which in turn improves executive function, while activity in brain regions such as the anterior cingulate gyrus, medial frontal gyrus, hippocampus, and dorsolateral prefrontal lobes is associated with anxiety (Ren et al., 2017; Li and Ouyang, 2021). Secondly, physical exercise can increase brain neuroendocrine levels and stimulate the secretion of dopamine, epinephrine, norepinephrine and other hormones in the brain (Cooper, 1973; Kruk et al., 2020), which are the main neurotransmitters affecting emotional states (Gosmann et al., 2021; Stubbendorff and Stevenson, 2021), thus improving individual executive function. Therefore, it has been proposed that executive function serves as a mediating role between physical activity and emotion improvement (Lin and Yu, 2015), but has not been validated by behavioral studies. In addition, for college students, each individual has different physical activity intensity and corresponding duration, so the impact of such differences in exercise habits on emotion and executive function still needs to be revealed by cross-sectional research.

Reviewing past studies, previous authors have more often explored the relationship between physical activity and anxiety and the effect of improving executive function, with the paucity of research on the mechanisms of how executive function plays a role between physical participation and the improvement of anxiety. Corresponding evidence can be found for the adverse effects of anxiety on each subcomponent of executive function, but the findings for the college student population remain divergent and require additional evidence from future research. Is executive function impaired in trait-anxious college students? Which specific sub-functions are impaired? How does physical activity level affect trait anxiety and executive function, and what is the relationship between the three? Do different intensity levels of exercise affect this correlation? Based on the above analysis, this study proposes the following hypotheses: (1) The inhibition function, working memory, and shifting function of college students with high trait anxiety are lower than those of college students with medium and low trait anxiety. (2) The level of physical activity was negatively correlated with trait anxiety. (3) Physical activity level of college students was positively correlated with executive function. (4) College students can enhance executive function through physical activity and consequently reduce trait anxiety, and the executive function plays a partially mediating role, and the paths and effects of different intensity levels of physical activity are not consistent.

Based on previous research results, the research team used cross-sectional study design, used one-way ANOVA, linear regression analysis, and structural equation modeling analysis to demonstrate each of the above hypotheses in an effort to discover whether the executive function is impaired in college students with trait anxiety, to clarify the underlying mechanisms by which physical activity level affects trait anxiety and the respective mediating roles of executive function subcomponents, to provide evidence at the behavioral level, so as to improve the theoretical system that physical activity improves cognitive and emotional states and to provide precise management solutions for college administrators.

## Participants and methods

### Participants

**Figure 1:**
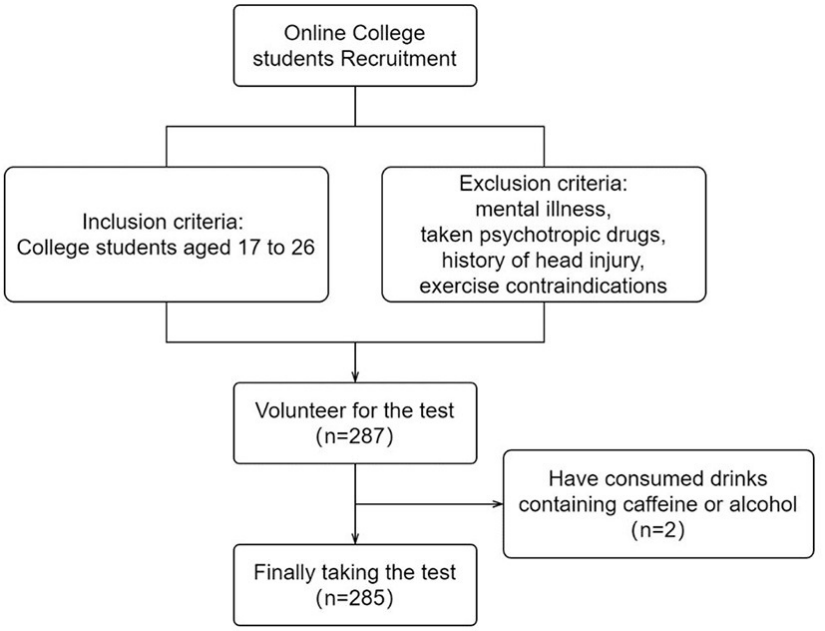
Flow chart for participant recruitment.

College students were recruited online under voluntary principles. Inclusion criteria were no mental illness, no previous use of barbiturates, benzodiazepines, or chloral hydrate, no strenuous exercise, and no caffeine or alcoholic beverages 24 h prior to testing. The study was approved by the Ethics committee of Shanghai University of Sport. The participant recruitment process is shown in Figure 1.

### Testing process

The study was conducted in December 2021. The tests were all conducted during the period of 13:30–16:30.

The questionnaire was distributed to the participants. Before filling out the questionnaire, the investigator read out the instructions and explained the contents, explaining that the data obtained were for scientific research only and emphasizing that the answers were true, independent, and voluntary. During the filling process, the participants were prompted to answer seriously according to the requirements. After completing the questionnaire, the investigator checked for missing items or answers contrary to common sense (e.g., age, height, and weight were outside the range of normal college students) and ensured that the information was complete by filling in the questionnaire again. Questionnaires with a filling time shorter than 2 min were excluded, trait anxiety questionnaires with five or more consecutive regular answers (e.g., 1,111, 2,222, 1,234, 4,321, etc.) were excluded, questionnaires with a total physical activity level of more than three times the standard deviation were excluded.

**Figure 2:**
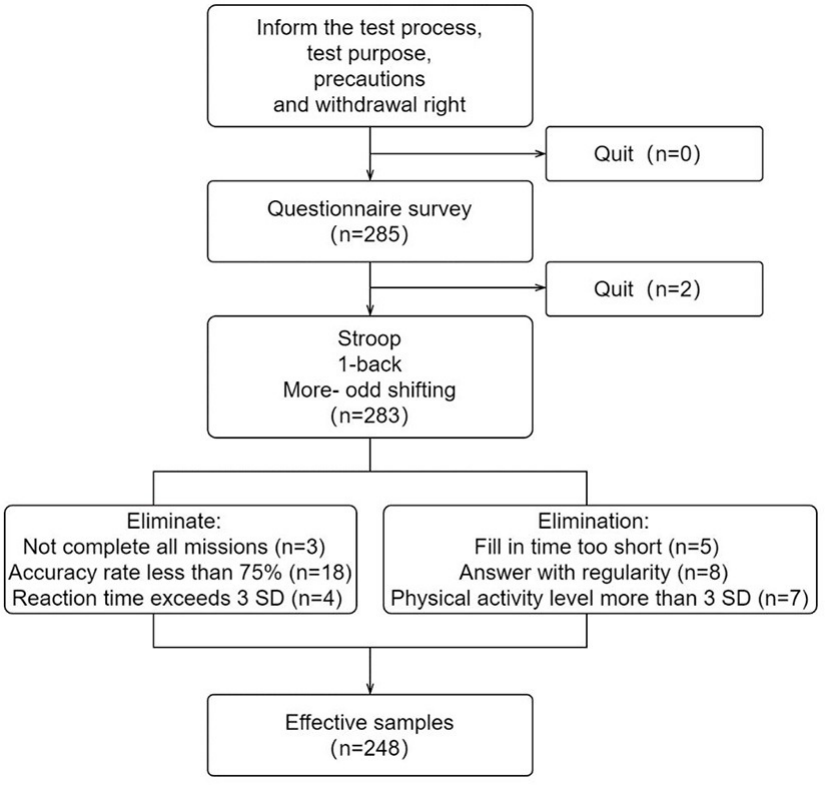
Flow chart for test process.

After that, three executive function tests are performed. Subjects were asked to press the key on their responses as soon as possible with the assurance of correctness and were informed that their test scores would be compared within the cluster to ensure subjective effort. Samples of executive function tasks with a correct rate of < 75% or a response time exceeding three times the standard deviation were excluded. The test process is shown in Figure 2.

### Measurement tools

#### State-trait anxiety inventory (STAI)

The State-trait Anxiety Inventory (STAI) was revised by Spielberger (Spielberger et al., 2017) and contained two subscales, the State Anxiety Inventory (S-AI) and the Trait Anxiety Inventory (T-AI). Only the Trait Anxiety Inventory was used in this study, containing 20 items, including 11 positive and nine negative scoring items. Four levels of scoring were used: “1” for almost never, “2” for somewhat, “3” for often, and “4” almost always. Scale scores were used as dependent variables in this study, high trait anxiety being one standard deviation above the mean score and low trait anxiety being one standard deviation below the mean score. A validation factor analysis of the scale showed χ/df = 3.90, TLl = 0.92, CFI = 0.94, RMSEA = 0.05, and SRMR = 0.05, indicating good construct validity. The scale Cronbach alpha coefficient was 0.81.

#### International physical activity questionnaire (IPAQ)

The International Physical Activity Questionnaire is one of the most valid and internationally accepted questionnaires for measuring physical activity levels in adults, containing seven questions that assess the subject's exercise over the past week. The questionnaire classified different physical activities into three intensities: vigorous physical activity (VPA), moderate physical activity (MPA), and light physical activity (LPA), with metabolic equivalent (MET) assigned to 8.0, 4.0, and 3.3, respectively. A certain intensity physical activity level = corresponding MET assignment × weekly frequency (day) × time per day (min). The total physical activity level was the sum of the three intensity physical activity. The Cronbach alpha coefficient for this scale was 0.90.

#### Executive function test

The executive function test was implemented by E-prime 3.0 software, with a computer screen refresh rate of 60Hz, *via* external keyboard keys.

The Stroop task evaluated the inhibition function. The stimulus materials were randomly presented Chinese characters “red,” “green,” and “blue” with different colors, and the presentation time was 1,500 ms, with a stimulus interval of 750 ms. Subjects were asked to judge the color of the Chinese characters, ignoring the word meaning interference, and the red, green, and blue words corresponded to the key of H, J, and K. The formal test was conducted for 48 trials.

1-back task assessed the working memory. Arabic numerals were presented one by one in the center of the screen, with a presentation time of 1,500 ms and a stimulus interval of 750 ms. Subjects were asked to carefully view and memorize the presented numerals and respond by pressing the F key if they were the same as the previous one or the L key if they were different. The formal test was divided into two segments of 25 trials each.

**Figure 3:**
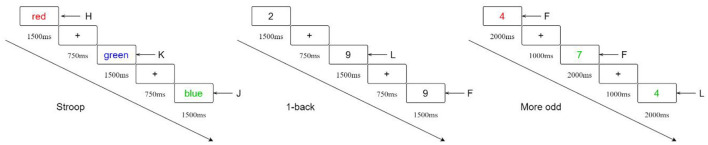
Schematic diagram of task paradigm.

A more-odd shifting task evaluated the shifting function. Arabic numbers were presented one by one in the center of the screen, with a presentation time of 2,000 ms and a stimulus interval of 1,000 ms. Subjects were asked to judge the numbers (1 to 9, but without 5). The first part was size judgment: After red numbers were presented, press F for numbers less than five and L for numbers greater than five. The second part was odd-even judgment: After green numbers were presented, press F for odd numbers and L for even numbers. The third part was the mixed judgment: the presented red number led to size judgment, and green led to odd-even judgment. The first and second parts had 16 trials each, and the third part had 32 trials. Schematic diagram of task paradigm are as detailed in Figure 3.

Each task had a practice session before the formal test. Based on previous studies, we subtracted the average reaction time between the incongruent and congruent conditions for inhibition, the average reaction time for working memory, and the average reaction time between the congruent and non-congruent conditions for shifting (Chen et al., 2011; Liu, 2017).

### Mathematical statistics

Measurement data conforming to normal distribution or approximate normal distribution were described by mean ± standard deviation, and comparison between groups was analyzed by one-way analysis of variance. Median (interquartile range) [M (P25, P75)] was used to describe measurement data with significantly skewed distribution, and Mann-Whitney U non-parametric test was used for comparison between groups. Count data were expressed as n (%), and intergroup comparisons were performed by chi-square analysis. Pearson correlation analysis and linear regression analysis were used to investigate the relationship between physical activity level, executive function, and trait anxiety. The Harman one-way test was used to test for common method bias effects, and structural equation models were developed to examine the role of executive function subcomponents in the relationship between physical activity level and trait anxiety (all variables were standardized before modeling). Model evaluation metrics were selected from RMR (root mean square residual), RMSEA (root mean square error of approximation), GFI (goodness of fit index), and NFI (normed fit index), CFI (comparative fit index) (Hou et al., 2006). The path analysis parameters were estimated using the non-parametric percentage bootstrap method (no strict requirements for the distribution of variables), the number of samples was set to 5,000, and the bias-corrected 95% confidence interval of the mediated path product did not cross 0 to define the mediating effect as statistically significant, with the Percentile 95% CI as a secondary indicator. All statistical inferences were tested by a two-tailed test, and the test level α was set at 0.05. *P* < 0.05, *P* < 0.01 and *P* < 0.001 were marked with “^*^,” “^**^” and “^***^,” all representing differences with statistical significance. One-way ANOVA, LSD *post-hoc* multiple testing, chi-square analysis, Pearson correlation analysis, Harman one-way test, and multiple linear regression analysis were performed with SPSS Statistics 23.0 software, and structural equation modeling, path analysis, and testing were performed with Amos 23.0 software.

## Results

### Executive functions and demographic characteristics of college students with different levels of trait anxiety

Based on previous studies, we divided trait anxiety into three groups (Ma et al., 2015), with high trait anxiety being one standard deviation above the mean score (39.931+9.161 ≈ 49); low trait anxiety being one standard deviation below the mean score (39.931–9.161 ≈ 31), and high, medium, and low trait anxiety scores were 52.805 ± 3.132, 40.795 ± 5.230, and 26.680 ± 2.559, respectively.

**Figure 4:**
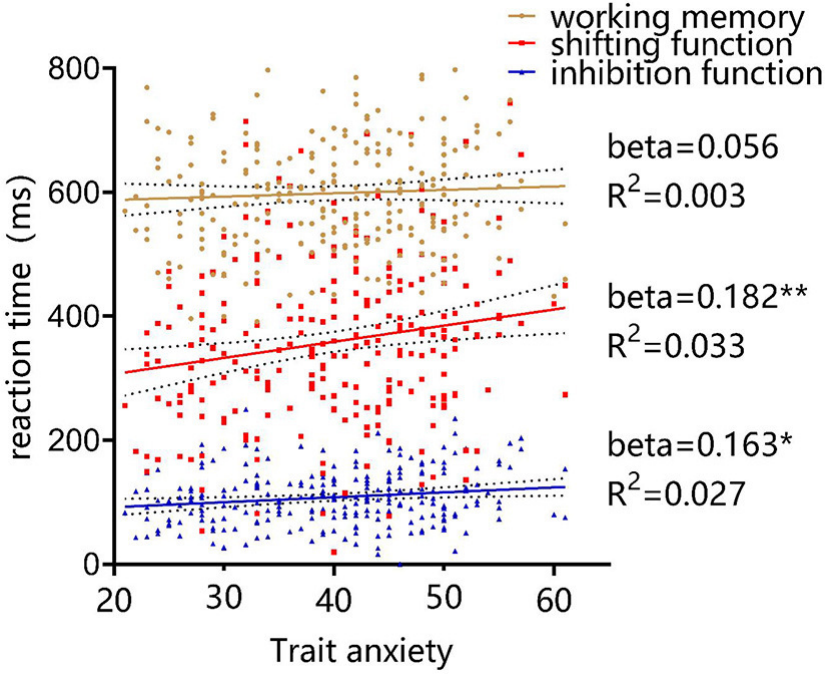
The relationship between trait anxiety and executive functions in college students. *P* < 0.05, *P* < 0.01 and *P* < 0.001 were marked with “*,” “**” and “***,” all representing differences with statistical significance.

Pearson bivariate correlation analysis showed that college students' trait anxiety scores were significantly correlated with shifting function (*r* = 0.182, *P* = 0.004) and inhibition function (*r* = 0.163, *P* = 0.010) and not with working memory (*r* = 0.056, *P* = 0.385), as shown in Figure 4. One-way ANOVA showed that the inhibition function of the low trait anxiety group was significantly better than that of the high trait anxiety group (*P* = 0.006), while the shifting function of the low trait anxiety group was significantly better than that of the medium trait anxiety group (*P* = 0.007) and the high trait anxiety group (*P* = 0.003), with no statistically significant difference in working memory between the three groups (*P* = 0.278). There were no significant differences in BMI, age, gender, and percentage of only children between the three groups (all *P* > 0.05), as detailed in Table 1. This result partially verified hypothesis 1 that college students with high trait anxiety have lower inhibition and conversion functions than those with low and medium trait anxiety but working memory did not show differences.

**Table 1:** Executive functions and demographic characteristics of college students with different levels of trait anxiety.

**Variables**	**Trait anxiety group**			**Comparison between groups**		***Post-hoc*** **multiple comparisons**		
	**Low (*n* = 50)**	**Medium (*n* = 156)**	**High (*n* = 41)**	***F*(χ^2^)**	***P*-value**	**L vs. M**	**L vs. H**	**M vs. H**
Trait anxiety (scores)	26.680 ± 2.559	40.795 ± 5.230	52.805 ± 3.132	386.514	0.000***	0.000***	0.000***	0.000***
PA (MET-min/week)	2068.920 ± 1241.478	1895.587 ± 1338.470	1219.451 ± 975.865	5.854	0.003**	0.400	0.002**	0.003**
VPA (min/week)	60 (0,127)	40 (0,90)	5 (0,75)	9.097	0.011*	0.099	0.004**	0.030*
MPA (min/week)	100 (57,180)	80 (15,150)	30 (0,95)	8.967	0.011*	0.205	0.002**	0.025*
LPA (min/week)	200 (149,300)	210 (140,385)	120 (90,275)	8.389	0.015*	0.440	0.017*	0.006**
Inhibition function (ms)	95.916 ± 41.805	108.033 ± 42.689	121.742 ± 51.588	3.866	0.022*	0.092	0.006**	0.078
Working memory (ms)	581.295 ± 94.451	601.124 ± 88.066	609.172 ± 87.997	1.286	0.278			
Shifting function (ms)	308.741 ± 106.073	366.268 ± 135.237	390.742 ± 130.149	5.279	0.006**	0.007**	0.003**	0.281
BMI (kg/m^2^)	21.362 ± 3.700	20.920 ± 3.550	21.344 ± 3.945	0.322	0.725			
Age (years)	20.083 ± 1.820	19.791 ± 1.830	19.564 ± 1.569	0.938	0.393			
Gender (male%)	0.533	0.423	0.455	1.642	0.440			
Only child (yes%)	0.644	0.556	0.563	1.618	0.806			

*P* < 0.05, *P* < 0.01 and *P* < 0.001 were marked with “*,” “**” and “***,” all representing differences with statistical significance.

### Relationship between physical activity and trait anxiety

**Figure 5:**
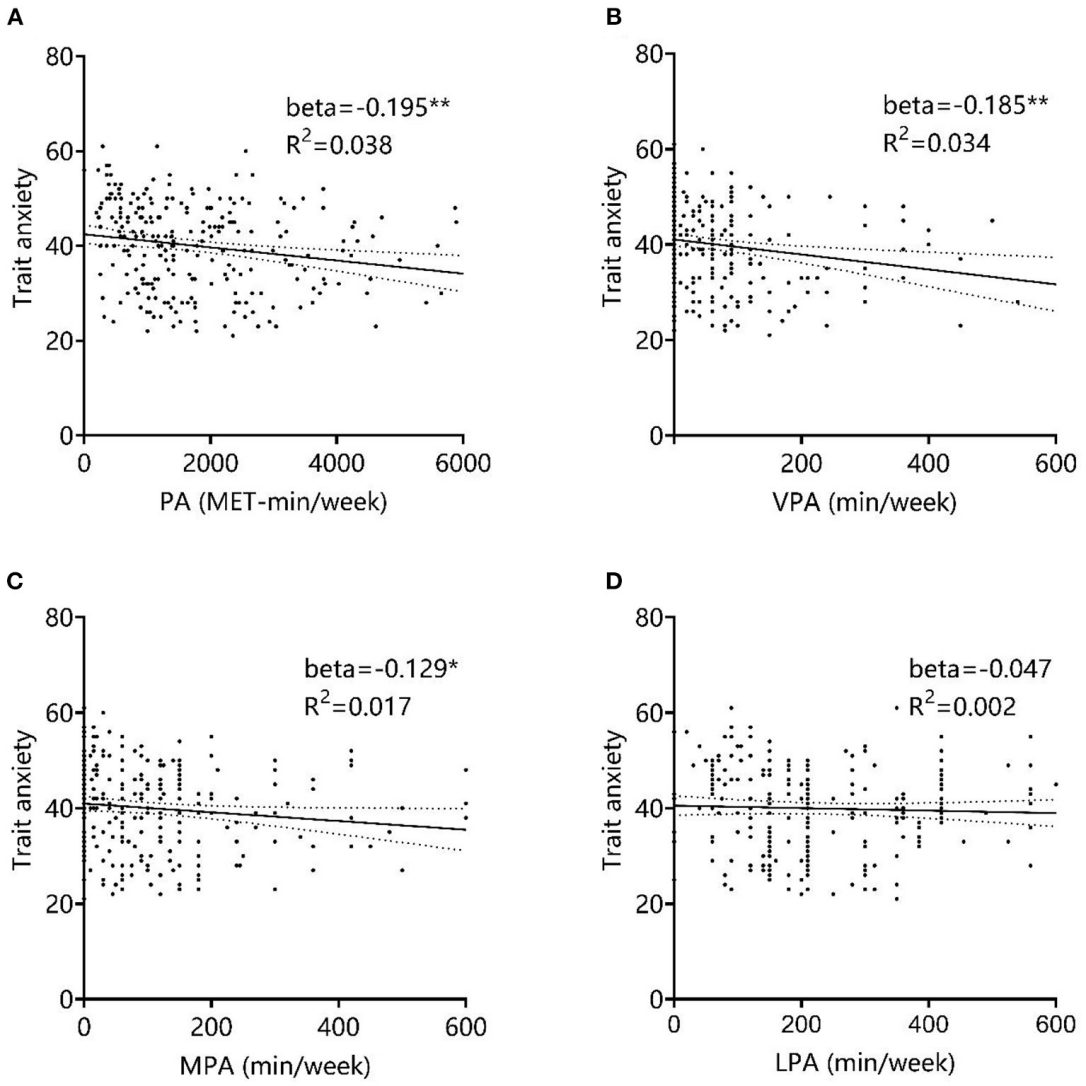
Effect of physical activity levels on trait anxiety. **(A)** PA, **(B)** VPA, **(C)** MPA, and **(D)** LPA. *P* < 0.05, *P* < 0.01 and *P* < 0.001 were marked with “*,” “**” and “***,” all representing differences with statistical significance.

Linear regression analysis was performed with physical activity level and different intensity levels of physical activity as independent variables and trait anxiety as the dependent variable, respectively. The results showed that total physical activity (Beta = −0.195, *P* = 0.002, *R*^2^ = 0.038), VPA (Beta = −0.185, *P* = 0.004, *R*^2^ = 0.034), and MPA (Beta = −0.129, *P* = 0.042, *R*^2^ = 0.017) all negatively predicted college students' trait anxiety levels, while the regression coefficients for LPA (Beta = −0.047, *P* = 0.463, *R*^2^ = 0.002) were not significant, as shown in Figure 5. This part of the results verified hypothesis 2, that higher levels of physical activity in college students are associated with lower levels of trait anxiety and that the best facilitation effect is seen with VPA.

### Relationship between physical activity and executive function

**Figure 6:**
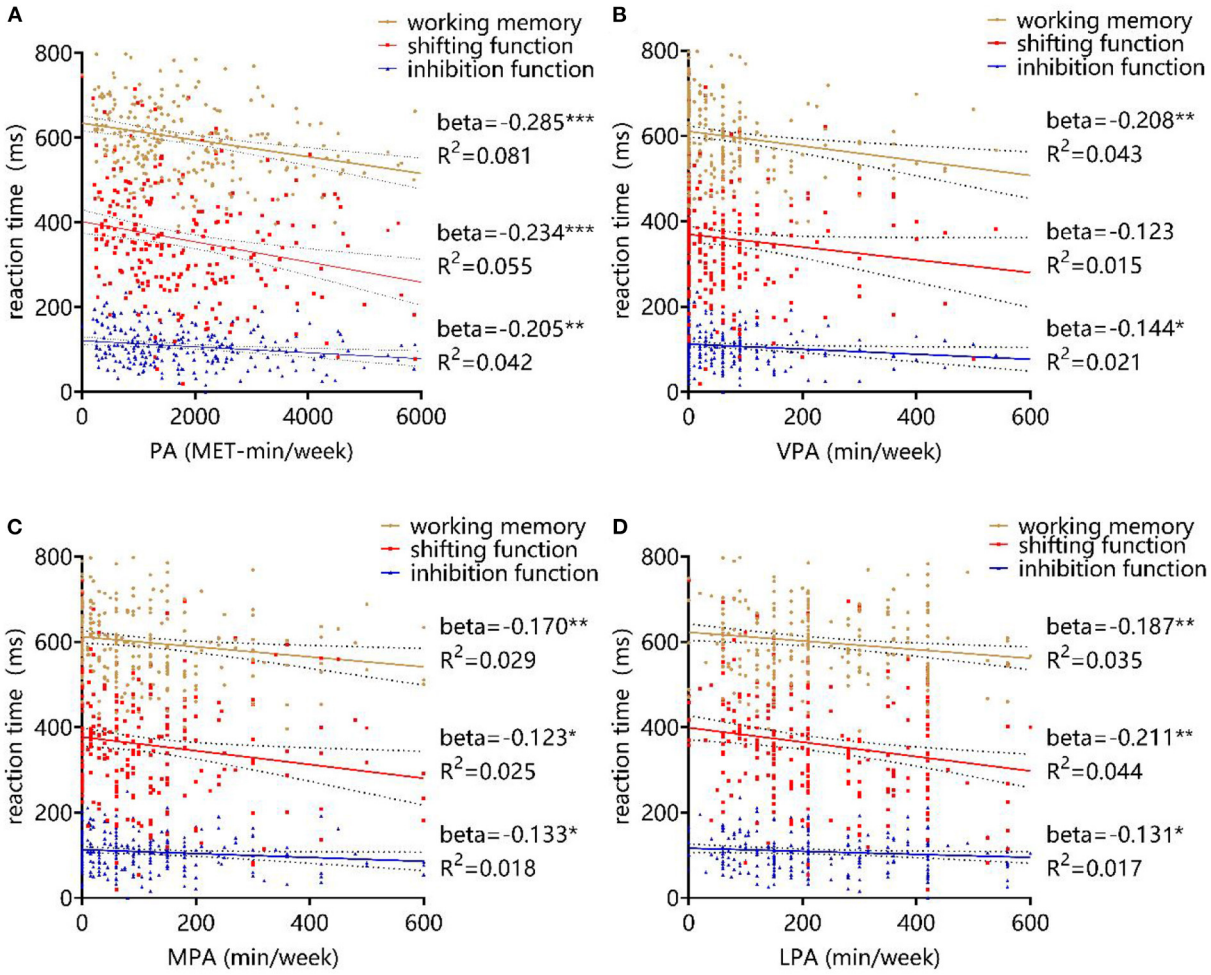
Effect of physical activity level on executive function. **(A)** PA, **(B)** VPA, **(C)** MPA, and **(D)** LPA. *P* < 0.05, *P* < 0.01 and *P* < 0.001 were marked with “*,” “**” and “***,” all representing differences with statistical significance.

Linear regression analysis was performed using the physical activity level and different intensity physical activity levels as independent variables and each subcomponent of executive functions as dependent variables, in separate cases. The results demonstrated that total physical activity (Beta = −0.285, *P* < 0.001, *R*^2^ = 0.081), VPA (Beta = −0.208, *P* = 0.001, *R*^2^ = 0.043), MPA (Beta = −0.170, *P* = 0.007, *R*^2^ = 0.029), LPA (Beta = −0.187, *P* = 0.003, *R*^2^ = 0.035) all negatively predicted working memory response time in college students. Physical activity level (Beta = −0.234, *P* < 0.001, *R*^2^ = 0.055), MPA (Beta = −0.159, *P* = 0.012, *R*^2^ = 0.025), and LPA (Beta = −0.211, *P* = 0.001, *R*^2^ = 0.044) all negatively predicted college students' shifting function effects, while the regression coefficient for the VPA (Beta = −0.123, *P*=0.053, R^2^=0.015) was not significant. Total physical activity (Beta = −0.205, *P* = 0.001, *R*^2^ = 0.042), VPA (Beta = −0.144, *P* = 0.024, *R*^2^ = 0.021), MPA (Beta = −0.133, *P* = 0.037, *R*^2^ = 0.018), and LPA (Beta = −0.131, *P* = 0.039, *R*^2^ = 0.017) all negatively predicted the amount of inhibition function effects in college students, as presented in Figure 6. This part of the results verified hypothesis 3 that higher levels of physical activity are associated with better executive function in college students, with VPA being the most beneficial in promoting working memory and inhibition function and LPA being the most beneficial in promoting shifting function.

### Construction and validation of a structural relationship model of physical activity level, executive function, and trait anxiety in college students

To examine the degree of influence of physical activity level and executive function on trait anxiety and to explore the feasibility of a structural relationship model, a multiple linear regression analysis (entry method) was conducted with trait anxiety score as the dependent variable and physical activity level, inhibition function, working memory, and shifting function as independent variables. The results indicated that the regression model passed the significance test [*F*_(4,243)_ = 4.882, *P* = 0.001, *R*^2^ = 0.075], and physical activity level (Beta = −0.156, *P* = 0.018), inhibition function (Beta = 0.147, *P* = 0.038), and shifting function (Beta = 0.135, *P* = 0.038) for the trait anxiety in college students were all significant predictors, while the regression coefficient for working memory (Beta = −0.081, *P* = 0.261) was not significant. The VIF values of each independent variable were all <5, and the effect of multicollinearity could be largely excluded from the results of this study. As detailed in Table 2.

**Table 2:** Multiple linear regression analysis of influencing factors of trait anxiety among college students.

**Dependent variable**	**Independent variables**	**B**	**SE**	**95%CI**		**Beta**	***t*-value**	**VIF value**	***F*-value**	**Model abstract**
				**Lower**	**Upper**					
Trait anxiety	Constant	40.264	4.520	31.450	49.129		8.909***		4.882**	*R*^2^ = 0.075
	Physical activity level	−0.001	0.000	−0.002	0.000	−0.156	−2.373*	1.138		ad*R*^2^ = 0.059
	Inhibition function	0.030	0.014	0.003	0.059	0.147	2.090*	1.296		
	Working memory	−0.008	0.007	−0.023	0.007	−0.081	−1.126	1.349		
	Shifting function	0.009	0.004	0.001	0.018	0.135	2.106*	1.078		

*P* < 0.05, *P* < 0.01 and *P* < 0.001 were marked with “*,” “**” and “***,” all representing differences with statistical significance.

To test for bias in common methods, a confirmatory factor analysis of the original entries and executive function performance of the International Physical Activity Questionnaire and Trait Anxiety Inventory was conducted using the Harman one-way test. Eight factors with characteristic roots >1 were obtained, and the variance explained by the first factor was 23.11%, which was much less than the critical value of 40%. Therefore, the effect of common method bias could be largely excluded from the results of this study.

**Figure 7:**
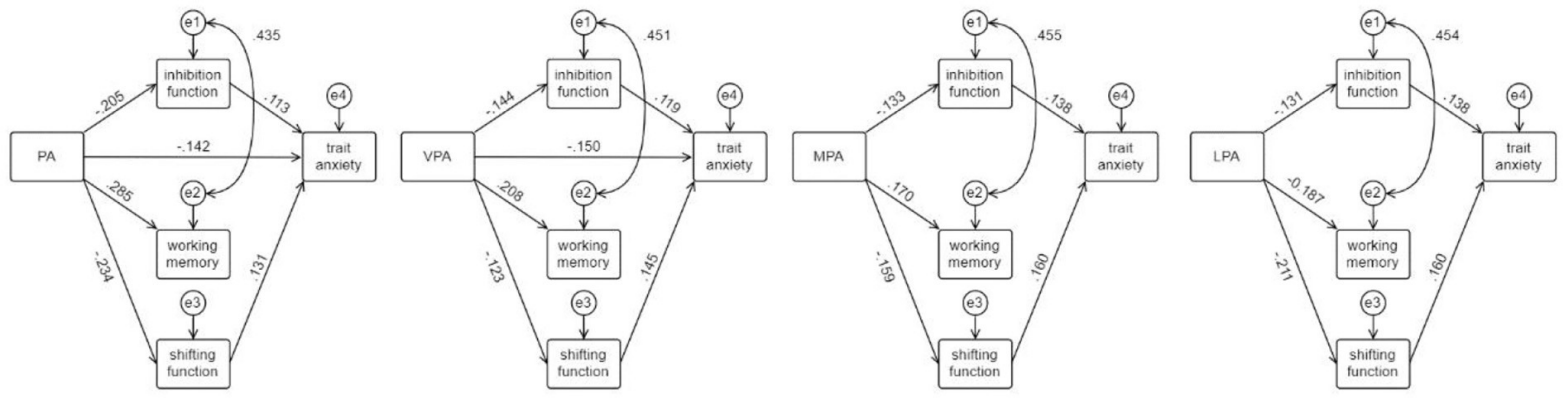
Schematic diagram of the pathway analysis of physical activity level affecting trait anxiety through executive function.

The research hypotheses were tested based on the interrelationship of physical activity level, executive function, and trait anxiety in college students. Model 1 was established with physical activity level as the independent variable, executive function as the mediating variable, and trait anxiety as the dependent variable, followed by three sub-models with high, medium, and low intensity physical activity levels as the independent variables, respectively. The paths with insignificant coefficients were removed one by one, and the path coefficients were recalculated until all passed the Bootstrap significance test. CMIN/df = 1.985 for model 1, indicating a good model fit, fully meeting the reference criteria of RMR < 0.05, RMSEA < 0.08, and GFI, NFI, and CFI values > 0.9, indicating a reasonable and reliable structural equation model. The CMIN/df of the three sub-models were all <3, and each goodness-of-fit index basically met the standard. The path analysis is shown in Figure 7, and the results of the mediating effect test are shown in Table 3.

**Table 3:** Results of bootstrap test for mediating effects.

**Model**	**Types of effects**	**Effect value B**	**Bootstrap SE**	**Bias-corrected 95%CI**	**Percentile 95%CI**	**Percentage of effects**	**Goodness of fit of the model**
Model 1	Total effect	−0.195**	0.059	[−0.313, −0.082]	[−0.314, −0.083]	100%	• CMIN/df = 1.985 • RMR = 0.042 • RMSEA = 0.063 • GFI = 0.990 • NFI = 0.951 • CFI = 0.973
	Direct effect	−0.141*	0.062	[−0.263, −0.019]	[−0.264, −0.020]	72.31%	
	Mediating effect of inhibition function	−0.023*	0.016	[−0.067, −0.001]	[−0.060, −0.001]	11.79%	
	Mediating effect of shifting function	−0.031*	0.017	[−0.076, −0.004]	[−0.069, −0.001]	15.90%	
Model 2	Total effect	−0.185**	0.066	[−0.326, −0.070]	[−0.332, 0.073]	100%	• CMIN/df = 2.892 • RMR = 0.054 • RMSEA = 0.088 • GFI = 0.986 • NFI = 0.916 • CFI = 0.939
	Direct effect	−0.150**	0.064	[−0.290, −0.040]	[−0.290, −0.040]	81.08%	
	Mediating effect of inhibition function	−0.017*	0.014	[−0.058, 0]	[−0.051, 0.002]	9.19%	
	Mediating effect of shifting function	−0.018*	0.013	[−0.054, −0.002]	[−0.048, 0]	9.73%	
Model 3	Total effect	−0.044**	0.019	[−0.091, −0.014]	[−0.087, −0.012]	100%	• CMIN/df = 2.496 • RMR = 0.057 • RMSEA = 0.078 • GFI = 0.984 • NFI = 0.898 • CFI = 0.932
	Direct effect						
	Mediating effect of inhibition function	−0.018*	0.013	[−0.054, −0.001]	[−0.049, 0]	40.91%	
	Mediating effect of shifting function	−0.025*	0.014	[−0.064, −0.005]	[−0.059, −0.002]	56.82%	
Model 4	Total effect	−0.052***	0.021	[−0.105, −0.019]	[−0.097, −0.017]	100%	• CMIN/df = 1.741 • RMR = 0.049 • RMSEA = 0.055 • GFI = 0.989 • NFI = 0.931 • CFI = 0.967
	Direct effect						
	Mediating effect of inhibition function	−0.018*	0.012	[−0.052, −0.002]	[−0.047, 0]	34.62%	
	Mediating effect of shifting function	−0.034**	0.017	[−0.076, −0.008]	[−0.071, −0.006]	65.38%	

*P* < 0.05, *P* < 0.01 and *P* < 0.001 were marked with “*,” “**” and “***,” all representing differences with statistical significance.

Physical activity level had a 72.31% direct effect on reducing trait anxiety (B = −0.195, 95% bootstrap CI: −0.313, −0.082), with the mediating effect of inhibition function accounting for 11.79% (B = −0.023, 95% bootstrap CI: −0.067, −0.001) and the mediating effect of shifting function accounting for 15.90% (B = −0.031, 95% bootstrap CI: −0.076, −0.004). VPA had a direct effect on the reduction of trait anxiety of 81.08% (B = −0.150, 95% bootstrap CI: −0.290, −0.040), with the mediating effect of inhibition function accounting for 9.19% (B = −0.017, 95% bootstrap CI: −0.058,0) and the mediating effect of shifting function of 9.73% (B = −0.018, 95% bootstrap CI: −0.054, −0.002). The effect of MPA on trait anxiety was fully mediated by executive function, with the mediating effect of inhibitory function accounting for 40.91% (B = −0.018, 95% bootstrap CI: −0.054, −0.001) and the mediating effect of shifting function accounting for 56.82% (B = 0.025, 95% bootstrap CI: −0.064, −0.005). The effect of LPA on trait anxiety was exclusively mediated by executive function, with 34.62% of the mediating effect mediated by inhibitory function (B = −0.018, 95% bootstrap CI: −0.052, −0.002) and 65.38% of the mediating effect mediated by shifting function (B = −0.034, 95% bootstrap CI: −0.076, −0.008). Different intensity levels of physical activity effectively improved working memory (B = −0.187 to −0.208) but working memory did not mediate between physical activity level and trait anxiety. This part of the results verified hypothesis 4.

## Discussion

The present study found that physical activity levels of college students were negatively associated with trait anxiety, supporting the findings of previous studies (He, 2016; Rogowska et al., 2020; Puccinelli et al., 2021). Some researchers have explored the mechanisms by which exercise improves mood that could explain this association. Neurobiological studies have found that physiological changes caused by exercise, such as decreased hypothalamic-pituitary-adrenal axis reactivity, increased cardiac natriuretic concentration, upregulated BDNF levels, increased neurocentral endorphins, downregulated postsynaptic 5-hydroxytryptamine receptors, increased irisin levels, increased hypothalamic temperature, hippocampal neurogenesis, and increased prefrontal alpha wave activity, are all associated with anxiety relief (DeBoer et al., 2012; Anderson and Shivakumar, 2013; Uysal et al., 2018; Kandola and Stubbs, 2020), suggesting that the anxiolytic exercise effects may be mediated by multiple physiological mechanisms. Social psychological research has found that self-esteem levels (Kayani et al., 2021), self-efficacy (Xu and Du, 2021), social support (Sabo et al., 2020) and other factors also play a mediating role in the positive impact of physical activity on mental health of college students. The results of this study provide new insights in this field, that is, the improvement of executive function (mainly inhibition and shifting function) may be the potential pathway for the improvement of anxiety symptoms after exercise. The study also found that VPA had the strongest association with anxiety, probably attributed to the fact that high-intensity physical activity provides more stimulation to increase body temperature and endorphin secretion (Jin et al., 2002; Gerber et al., 2014). It is worth noting that the physical activity intensity in the scale relies on self-perception rather than physiological indicators, which may explain some inconsistent findings, but it suggests that the direct effect of trait anxiety reduction requires adequate exercise load stimulation. While the effect of light and moderate intensity physical activity on trait anxiety was fully mediated by executive functions, where the mediating effect of the shifting function was higher than that of the inhibition function, suggesting that physical activity levels reduce trait anxiety with multiple pathways of effects and that exercise program design needs to take different intensities and durations into account, with some structural effects.

The present study found that physical activity had different effects on each subcomponent of executive function. The effects in descending order were working memory, shifting function, and inhibition function, with VPA being the most favorable for promoting working memory and inhibition function and LPA being the most favorable for promoting shifting function, which is basically consistent with the findings of Chen et al. (2011). In contrast, Liu (2017) found that aerobic exercise had the most significant effect on college students' working memory promotion, with inhibition function and shifting function second. The inconsistent conclusions may be explained by the fact that Liu Junyi chose a simpler and single form of aerobic physical exercise, which only required the completion of a few simple prescribed movements, and the effect of the exercise form on shifting function was smaller. A large amount of research has been conducted to demonstrate beneficial effects of sports participation on various executive function subcomponents. Past studies found that exercise elevates peripheral brain-derived neurotrophic factor and insulin-like growth factor 1 levels, increases vascular endothelial growth factor and serotonin concentrations and stimulates hippocampal neurogenesis, promotes the volume of hippocampus, gray matter and white matter, improves the integrity of white matter, and increases the functional connectivity of frontal executive network, thus promoting executive function (Stillman et al., 2016; Marinus et al., 2019; Jiang et al., 2020). However, “dose-effect” and “selective effect” are still in the exploratory stage, which marks the precision of the research field and prompts the academy to further integrate the evidence from biological and behavioral studies.

The critical finding of this study is that physical activity could indirectly affect trait anxiety by improving executive function. It was found that individuals with high trait anxiety had enhanced activity in the dorsolateral prefrontal lobe and diminished connections with the posterior lateral frontal lobe, dorsolateral amygdala, and left syrinx gyrus. These results indicated that their nervous system had reduced processing efficiency, which led to reduced attentional control system function (Basten et al., 2011), and that their enhanced dorsolateral prefrontal activity might be related to their attempts to compensate for the impairment in attentional control function, which further supports the attentional control theory proposed by Eysenck et al. (2007). And cognitive neural model suggests that high-anxiety individuals have reduced attentional control involvement from the top-down, resulting in insufficient prefrontal attentional control activation (Bishop, 2009). In addition, individuals with executive function deficits have poor emotion regulation (Landis et al., 2021), and cognitive training helps college students relieve anxiety (Callinan et al., 2015), both of which may have bidirectional effects. Given the close relationship between anxiety and executive functions and the overlap between brain mechanisms related to executive function and emotion activated by exercise, it is necessary to include an examination of executive function in the study of mechanisms of exercise for mental health.

The significance of this study is that it provides the first insight into the relationship between physical activity level, executive function, and emotion improvement mediated by executive function, which also clarifies the effect of different intensity physical activity levels on improving trait anxiety. The findings not only reveal the importance of executive function in the study of physical activity and emotion but also further rationalize the behavioral mechanisms by which exercise promotes mental health.

## Limitations and prospects

(1) This study used a cross-sectional design with limited ability to make causal inferences. More longitudinal studies are recommended in the future to explore further the relationship between temporal changes in physical activity level, executive function, and trait anxiety and the associated effect sizes.
(2) This study only examined the intensity and duration of physical activity levels and was measured using a self-report questionnaire, which may have some subjective bias. It is recommended that future studies combine exercise form and frequency and use measurement tools such as accelerometers and heart rate bands to collect information from subjects for more accurate reporting of physical activity levels.
(3) College students' sports participation habits and mental health status are inevitably influenced by the social environment during the same period. Whether the findings of this study can be generalized to other groups of people remains for further analysis and comparison.

## Conclusion and suggestions

College students with trait anxiety suffer from impaired inhibition and shifting function. The facilitation effects of physical activity levels on executive function subcomponents were, in descending order, working memory, shifting function, and inhibition function, with VPA contributing most to the facilitation of working memory and inhibition function, and LPA contributing most to the promotion of shifting function. Physical activity promotes both inhibition and shifting functions, which in turn affect trait anxiety. VPA has a direct effect on trait anxiety, while the anxiolytic effect of MPA and LPA are mediated exclusively through executive functions, with the highest mediating effect of shifting function. It is recommended that college workers should motivate students with high trait anxiety to engage in more VPA and pay attention to changes in their inhibition and shifting functions.

## Data availability statement

The original contributions presented in the study are included in the article/supplementary material, further inquiries can be directed to the corresponding author/s.

## Author contributions

ZD: data collection and analysis. PW: writing the original draft and executive function test. XX, JW, and JZ: data curation. XW: review and editing. All authors contributed to the article and approved the submitted version.

## Funding

This work was supported financially by the Key Laboratory Project of Shanghai Science and Technology Commission (Grant No. 11DZ2261100).

## Conflict of interest

The authors declare that the research was conducted in the absence of any commercial or financial relationships that could be construed as a potential conflict of interest.

## Publisher's note

All claims expressed in this article are solely those of the authors and do not necessarily represent those of their affiliated organizations, or those of the publisher, the editors and the reviewers. Any product that may be evaluated in this article, or claim that may be made by its manufacturer, is not guaranteed or endorsed by the publisher.
